# Heritability of Antibody Isotype and Subclass Responses to *Plasmodium falciparum* Antigens

**DOI:** 10.1371/journal.pone.0007381

**Published:** 2009-10-08

**Authors:** Nancy O. Duah, Helen A. Weiss, Annette Jepson, Kevin K. A. Tetteh, Hilton C. Whittle, David J. Conway

**Affiliations:** 1 Department of Infectious and Tropical Diseases, London School of Hygiene and Tropical Medicine, London, United Kingdom; 2 Medical Research Council Laboratories, Fajara, The Gambia; BMSI-A*STAR, Singapore

## Abstract

**Background:**

It is important to understand the extent to which genetic factors regulate acquired immunity to common infections. A classical twin study design is useful to estimate the heritable component of variation in measurable immune parameters.

**Methodology/Principal Findings:**

This study assessed the relative heritability of different plasma antibody isotypes and subclasses (IgG1, IgG2, IgG3, IgG4, IgM, IgA and IgE) naturally acquired to *P. falciparum* blood stage antigens AMA1, MSP1-19, MSP2 (two allelic types) and MSP3 (two allelic types). Separate analyses were performed on plasma from 213 pairs of Gambian adult twins, 199 child twin pairs sampled in a dry season when there was little malaria transmission, and another set of 107 child twin pairs sampled at the end of the annual wet season when malaria was common. There were significantly positive heritability (*h* ^2^) estimates for 48% (20/42) of the specific antibody assays (for the seven isotypes and subclasses to the six antigens tested) among the adults, 48% (20/42) among the children in the dry season and 31% (13/42) among the children in the wet season. In children, there were significant heritability estimates for IgG4 reactivity against each of the antigens, and this subclass had higher heritability than the other subclasses and isotypes. In adults, 75% (15/20) of the significantly heritable antigen-specific isotype responses were attributable to non-HLA class II genetic variation, whereas none showed a significant HLA contribution.

**Significance:**

Genome-wide approaches are now warranted to map the major genetic determinants of variable antibody isotype and subclass responses to malaria, alongside evaluation of their impact on infection and disease. Although plasma levels of IgG4 to malaria antigens are generally low, the exceptionally high heritability of levels of this subclass in children deserves particular investigation.

## Introduction

Genetic variation among individuals profoundly affects susceptibility to clinical malaria, although only a minority of the causal polymorphisms are known [1], [2]. It is particularly important to identify genetic determinants of naturally acquired immune responses, as discovering the underlying molecular mechanisms could lead to better vaccine design and use [3]. In Africa, there are some ethnic differences in naturally acquired immune responses and resistance to malaria [4]–[6]. However, most of the variation in the human genome exists within each population, and remains unexamined for effects on immune responses.

An estimate of the heritability of a phenotypic trait can be derived by a Classical Twin Study (CTS) [7]. A CTS determines whether concordance of a trait among people with a relatively shared common environment is attributable mainly to genetic factors, and studies monozygous (MZ) ‘identical’ twins from a single fertilised ovum and dizygous (DZ) ‘non-identical’ twins from separate fertilised ova who are genetically related as normal siblings sharing ∼50% of parental alleles [8]. The genetic contribution to the overall variance in a phenotype can be estimated by comparing the intra-pair similarity of monozygous twins (MZ) and dizygous twins (DZ) [9], thus deriving an estimate of heritability (*h* ^2^) [10]. Such findings may be generalised to populations if the trait is normally the same in twins and singletons, and if it has no association with zygosity or gender [7].

An early study of twins in Liberia indicated that antibody levels to the *Plasmodium falciparum* ring-infected erythrocyte surface antigen (RESA) were influenced by non-HLA genes [11]. This finding was consistent with a lack of association observed between HLA alleles and antibody levels to this antigen in The Gambia or Madagascar [12]. A study of adult twins in The Gambia indicated significant heritability of cell mediated and antibody responses to *P. falciparum* antigens, with the apparent contribution of HLA to this being variable among assays and antigens tested [13]. By comparison, a family-based study in Papua New Guinea indicated heritable effects on antibody responses to *P. falciparum* antigens to be generally non-HLA-linked [14].

The basis of clinically relevant differences between individuals in antibody class and IgG subclass response polarisation needs to be investigated. For example, cytophilic IgG1 and IgG3 antibodies enable phagocytosis [15] and antibody dependent cellular inhibition of parasites within erythrocytes [16], and are more commonly associated with protection from malaria [17]–[19], while IgG2 and IgG4 subclasses lack such activity and might in some cases block cytophilic antibodies [20]. Production of antibody classes and subclasses is influenced by different cytokines, including interferon gamma (IFNγ), interleukin 4 (IL-4) and IL-5 from helper T cells, IL-10 from regulatory T cells, and transforming growth factor beta (TGFβ) from macrophages and regulatory T cells [21]–[26]. The current study was designed to investigate the role of genetic variation in determining the acquisition of all naturally acquired plasma antibody isotypes and subclasses (IgG1, IgG2, IgG3, IgG4, IgM, IgA and IgE), to a panel of several *P. falciparum* blood stage antigens which are considered to be vaccine candidates. The study employed samples from Gambian adult and child twins that were previously assayed for IgG and IgM with a small number of antigens. The aim was to estimate and compare the heritabilities of all the antibody isotype responses, to test whether these heritability estimates varied according to whether children were sampled during the peak or minimal malaria transmission periods, and to evaluate the influence of HLA class II and non-HLA genes.

## Materials and Methods

### Ethics statement

The study of genetics of immune responses to malaria in the adult and child twins was reviewed and approved by the MRC Gambia Scientific Co-ordinating Committee and the Gambia Government/MRC Joint Ethics Committee (the ethics committee based in The Gambia that reviews all proposals in the country). At the time of approval in 1991, and during subsequent recruitment in 1991–1993, verbal informed consent was the standard practice for observational studies in The Gambia, reflecting the low literacy rate in the general population at that time, a practice guided by over 40 years of medical research experience which was found culturally appropriate and acceptable to observational study subjects. The purpose of the study was explained to potential participants in local languages and all subjects or both of their parents gave verbal informed consent. More recently, literacy rates have increased substantially and written informed consent has become standard practice for research, as incorporated in 2000 into the guidelines for the Gambia Government/MRC Joint Ethics Committee. The proposal for investigation of antibody isotypes in the samples presented here was further reviewed and approved by both the MRC Gambia Scientific Co-ordinating Committee and the Gambia Government/MRC Joint Ethics Committee in 2006.

### Plasma samples from adult and child twins

The plasma samples were prepared from heparinised blood samples (between 5 and 10 ml) collected from subjects living in malaria endemic areas of The Gambia between 1991 and 1993, and consist of three groups. The first group comprises 213 pairs of adult twins (58 monozygous and 155 dizygous) with a mean age of 27.0 years (range 14–92 years) sampled between January 1992 and May 1993. The second group comprises 199 pairs of child twins (32 monozygous and 167 dizygous) with a mean age of 5.0 years (range 1–10 years) sampled at the end of an annual dry season in April – May 1991 when there was minimal or no malaria transmission. The third group comprises 107 pairs of child twins (30 monozygous and 77 dizygous) with a mean age of 5.7 years (range 1–10 years) sampled at the end of the wet season (October - November 1991) when malaria incidence was highest. The children in the third group (wet season sample) were a subset of those in the second group (dry season sample). There were no significant differences between the mean ages of MZ and DZ twins for any of the three groups. The study sites and populations are described previously, when more limited analyses of antibody reactivities to malaria antigens were investigated [27], [28]. Plasma samples were stored at −40°C for a period of approximately 15 years with no inadvertent thawing prior to the assays described here.

### *P. falciparum* blood stage antigens

The antigens used for assays were *Escherichia coli* expressed recombinant proteins. The apical membrane antigen 1 (AMA1) recombinant protein represented the full length ectodomain containing amino acids 83–531 of the AMA1 sequence in the 3D7 clone [29]; for the 19-kDa C-terminal fragment of merozoite surface protein 1 (MSP1-19), a glutathione s-transferase (GST) fusion protein represented amino acids 1631–1726 of the MSP1 sequence in the Wellcome isolate [30]; the two MSP2 allelic antigens used were GST fusion proteins representing MSP2 amino acid positions 1–184 of the ch150/9 isolate and 22–247 of the Dd2 clone [31], [32] (here termed MSP2A and MSP2B, respectively); the two MSP3 allelic antigens represented the MSP3 amino acids 2–354 of the 3D7 clone and 2–379 of the K1 isolate [33] (here termed MSP3A and MSP3B, respectively).

### Antibody isotype ELISA assays

Enzyme-linked immunosorbent assays (ELISAs) were used to detect plasma antibody isotypes IgG1, IgG2, IgG3, IgG4, IgM, IgA and IgE to the panel of *P. falciparum* blood stage antigens AMA1, MSP1-19, MSP2-ch150/9 (MSP2A), MSP2-Dd2 (MSP2B), MSP3-3D7 (MSP3A) and MSP3-K1 (MSP3B), following protocols similar to those previously described [31], [33]–[35]. Briefly, wells of flat bottom 96-well plates (Immulon 4HBX, ThermoLabs) were coated with 50 ng of recombinant antigen in 100 µl of coating buffer (15 mM Na_2_CO_3_, 35 mM NaHCO_3_, pH 9.3), incubated at 4°C overnight and then washed 4 times with washing buffer (PBS with 0.05% Tween 20). Unoccupied binding sites were blocked with 200 µl per well of blocking buffer (1% skimmed milk, Marvel^TM^, UK, in washing buffer) for 5 hours at room temperature. Plates were washed 4 times prior to the addition of 100 µl plasma diluted with blocking buffer (1/500 for IgG1 and IgG3; 1/50 for IgG2, IgG4, IgM and IgA, 1/10 for IgE), these dilutions were chosen after optimisation of assays for each isotype. After incubation at 4°C overnight the plates were washed 4 times, and 100 µl of horseradish peroxidase-conjugated anti-human IgG subclasses (anti-IgG1-4 murine monoclonals AP006-AP009, The Binding Site, UK), anti-IgM (P0215, Dako, Denmark), anti-IgA (P0216, Dako) at 1/5000, or anti-IgE (P0295, Dako) at 1/2000 was added to each well and incubated at room temperature for 3 hours. Plates were washed 4 times and antibody reactivities detected with 100 µl of substrate (0.4mgml-1 of *o*-phenylenediamine; 0.08% H_2_O_2_) in developing buffer (24.5 mM citric acid monohydrate; 52 mM Na_2_HPO_4_, pH 5.0). The reaction was stopped with 50 µl of 2 M H_2_SO_4_ and optical density (OD) of plates measured at 490 nm. The OD reading was corrected for non-specific binding by subtracting the OD reactivity to the maltose-binding protein (MBP) fusion tag for MSP3, and glutathione s-transferase (GST) fusion tag for MSP1 and MSP2 recombinant proteins. All assays were performed with each plasma sample tested in duplicate wells, and the mean OD value determined. Each pair of twin plasma was run on the same plate to avoid plate-to-plate variations in measurements.

### Data analysis

Antibody data generated by ELISA in the form of OD values were log transformed after adding a value of 1, and transferred from Excel spreadsheets to SPSS version 11 (SPSS Inc., Chicago, IL) and GraphPad Prism version 4.0 (GraphPad Software, San Diego, CA) for analysis. Pairwise correlations using Pearson's correlation coefficient were performed for MZ and DZ twins separately. The Mx Graphical User interface [GUI] version 1.3.65 [Virginia, VA] [36], [37] was used for the estimation of heritabilities. This package gives the heritabilities and confidence intervals as well as the P values for statistical differences between the additive genetic and the common and unique environments, based on maximum likelihood estimation [36]. For this study we used the ACE model (A, the average effects of genes influencing the phenotype; C, environmental effects shared within families and giving resemblance among family members; E, environmental effects on an individual that do not contribute to resemblance among family members) because it gives a conservative estimate of heritability compared to other models.

The zygosities of the adult and child twins had been ascertained using minisatellite typing with 5 minisatellite probes, and HLA typing performed using Southern blot hybridisation with radiolabelled HLA-DRB and HLA-DQB probes, as described in previous studies on the samples [13], [27]. The relative contributions of HLA class II and non-HLA class II genes to the observed genetic variance was assessed by the comparison of HLA DRB-DQB identical MZ and DZ for non-HLA gene contributions and HLA identical versus HLA non-identical DZ for HLA gene contributions. Using data from all of the antigens, Wilcoxon signed ranks tests were applied to examine overall differences in heritabilities between antibody isotypes, within each of the studies of adults or children, and differences in the distribution of heritability estimates from adults and children.

## Results

**Figure 1:**
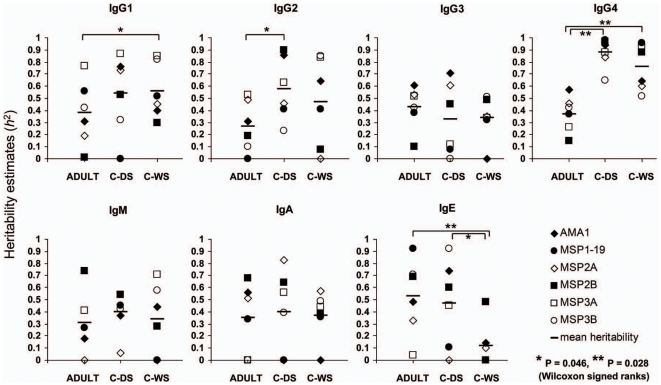
Heritability of antibody isotype levels to *P. falciparum* blood stage antigens in Gambian adults (213 twin pairs), children in the dry season (C-DS, 199 twin pairs) and children in the wet season (C-WS, 107 twin pairs). Each symbol represents the point estimate of heritability (*h* ^2^) for each isotype or subclass against each antigen. Significant differences in *h* ^2^ estimates between analyses for adults and children are shown (P values of 0.05 or less with Wilcoxon signed ranks tests).

The set of seven antibody isotypes and subclasses against the panel of six *P. falciparum* blood stage antigens, was measured for all 1038 plasma samples from the adult and child twins. The analyses on heritability are shown separately from each of the three different studies, on the 213 adult twin pairs in Table 1, the 199 child twin pairs in the dry season in Table 2, and the 107 child twin pairs in the wet season in Table 3. As expected, in each of the analyses the observed correlations of the isotype levels were generally higher in the MZ than the DZ twins reflecting a degree of genetic regulation. There were significant heritability estimates for 48% (20/42), 48% (20/42) and 31% (13/42) of the antigen-specific antibody isotypes for adults, children in the dry season and children in the wet season respectively. The slightly lower proportion of significant estimates in the children in the wet season is due to the smaller sample size, as the point estimates of heritability were equally high in this group as in the children studied in the dry season (Figure 1, and a comparison of Tables 2 and 3).

**Table 1:** Pairwise correlations (*r*) and heritability estimates (*h* ^2^) of antibody isotype responses to *P. falciparum* blood stage antigens for adult twins.

Antibody	Antigens	Monozygous *r* (n = 58)	Dizygous *r* (n = 155)	Heritability *h* ^2^	95% CI	P value
IgG1	AMA1	0.70	0.56	0.31	0.0–0.59	**0.04**
	MSP1-19	0.61	0.35)	0.56	0.14–0.74	**0.01**
	MSP2A	0.39	0.27	0.19	0.0–0.55	0.46
	MSP2B	0.19	0.18	0.01	0.0–0.40	0.97
	MSP3A	0.77	0.34	0.77	0.53–0.84	**0.0001**
	MSP3B	0.68	0.48	0.42	0.06–0.74	**0.02**
IgG2	AMA1	0.64	0.36	0.31	0.0–0.66	0.17
	MSP1-19	0.07	0.13	0	0.0–0.24	-
	MSP2A	0.70	0.39	0.49	0.10–0.73	**0.01**
	MSP2B	0.49	0.43	0.19	0.0–0.59	0.42
	MSP3A	0.64	0.27	0.53	0.10–0.68	**0.01**
	MSP3B	0.52	0.30	0.10	0.0–0.53	0.67
IgG3	AMA1	0.53	0.36	0.61	0.15–0.76	**0.01**
	MSP1-19	0.32	0.17	0.38	0.0–0.57	0.09
	MSP2A	0.72	0.39	0.53	0.16–0.76	**0.006**
	MSP2B	0.56	0.50	0.10	0.0–0.44	0.67
	MSP3A	0.62	0.32	0.52	0.10–0.71	**0.01**
	MSP3B	0.61	0.40	0.42	0.0–0.72	0.05
IgG4	AMA1	0.44	0.12	0.57	0.24–0.78	**0.007**
	MSP1-19	0.05	0.21	0.37	0.0–0.58	0.34
	MSP2A	0.58	0.38	0.46	0.03–0.72	**0.03**
	MSP2B	0.52	0.46	0.15	0.0–0.53	0.48
	MSP3A	0.32	0.19	0.26	0.0–0.50	0.37
	MSP3B	0.49	0.18	0.42	0.0–0.59	0.05
IgM	AMA1	0.45	0.34	0.18	0.0–0.58	0.46
	MSP1-19	0.60	0.55	0.27	0.0–0.58	0.14
	MSP2A	0.61	0.65	0	0.0–0.28	-
	MSP2B	0.72	0.36	0.74	0.46–0.82	**0.0001**
	MSP3A	0.58	0.50	0.41	0.02–0.74	**0.03**
	MSP3B	0.25	0.32	0.27	0.0–0.62	0.41
IgA	AMA1	0.55	0.19	0.56	0.37–0.71	**0.002**
	MSP1-19	0.34	0.16	0.34	0.0–0.52	0.16
	MSP2A	0.65	0.18	0.51	0.20–0.68	**0.008**
	MSP2B	0.54	0.35	0.68	0.31–0.78	**0.002**
	MSP3A	0.15	0.27	0	0.0–0.34	-
	MSP3B	0.11	0.37	0	0.0–0.19	-
IgE	AMA1	0.43	0.07	0.48	0.04–0.81	**0.03**
	MSP1-19	0.66	0.01	0.92	0.84–0.95	**0.001**
	MSP2A	0.45	0.14	0.33	0.0–0.48	0.19
	MSP2B	0.50	0.28	0.69	0.45–0.81	**0.001**
	MSP3A	0.10	0.03	0.04	0.0–0.27	0.82
	MSP3B	0.47	0.09	0.71	0.26–0.87	**0.006**

*r*, Pearson's correlation coefficient; n, number of twin pairs; CI, confidence interval of *h* ^2^ estimate; P values for significant heritability estimates are in bold. Analysis for IgE was performed with 56 Monozygous and 138 Dizygous twin pairs.

**Table 2:** Pairwise correlations (*r*) and heritability estimates (*h* ^2^) of antibody isotype responses to *P. falciparum* blood stage antigens for child twins (dry season).

Antibody	Antigens	Monozygous *r* (n = 32)	Dizygous *r* (n = 167)	Heritability *h* ^2^	95% CI	P value
IgG1	AMA1	0.83	0.50	0.76	0.52–0.92	**0.0001**
	MSP1-19	0.34	0.34	0	0.0–0.53	-
	MSP2A	0.73	0.46	0.73	0.39–0.88	**0.001**
	MSP2B	0.75	0.59	0.53	0.13–0.87	**0.01**
	MSP3A	0.81	0.40	0.87	0.67–0.92	**0.0001**
	MSP3B	0.51	0.39	0.32	0.0–0.71	0.27
IgG2	AMA1	0.85	0.44	0.86	0.59–0.92	**0.0001**
	MSP1-19	0.78	0.09	0.41	0.10–0.62	**0.02**
	MSP2A	0.71	0.49	0.46	0.0–0.79	0.06
	MSP2B	0.86	0.36	0.90	0.71–0.95	**0.0001**
	MSP3A	0.72	0.28	0.63	0.24–0.77	**0.007**
	MSP3B	0.60	0.40	0.23	0.0–0.65	0.35
IgG3	AMA1	0.65	0.40	0.71	0.28–0.84	**0.006**
	MSP1-19	0.06	0.03	0.08	0.0–0.40	0.73
	MSP2A	0.71	0.49	0.61	0.24–0.86	**0.005**
	MSP2B	0.70	0.56	0.45	0.0–0.83	**0.06**
	MSP3A	0.42	0.40	0.12	0.0–0.61	0.69
	MSP3B	0.30	0.39	0	0.0–0.30	-
IgG4	AMA1	0.84	0.56	0.94	0.86–0.97	-
	MSP1-19	0.67	0.08	0.98	0.94–0.99	-
	MSP2A	0.82	0.27	0.84	0.66–0.91	**0.0001**
	MSP2B	0.95	0.36	0.97	0.84–0.98	**0.0001**
	MSP3A	0.62	0.17	0.88	0.52–0.94	**0.001**
	MSP3B	0.61	0.21	0.65	0.31–0.83	**0.006**
IgM	AMA1	0.76	0.57	0.37	0.0–0.66	0.05
	MSP1-19	0.55	0.16	0.45	0.03–0.64	**0.04**
	MSP2A	0.24	0.31	0.06	0.0–0.60	0.88
	MSP2B	0.48	0.27	0.54	0.0–0.74	0.14
	MSP3A	0.67	0.34	0.54	0.01–0.75	0.05
	MSP3B	0.68	0.44	0.42	0.0–0.76	0.09
IgA	AMA1	0.21	0.32	0	0.0–0.18	-
	MSP1-19	0.68	0.52	0	0.0–0.10	-
	MSP2A	0.65	0.45	0.83	0.53–0.91	**0.0001**
	MSP2B	0.47	0.22	0.64	0.10–0.84	**0.03**
	MSP3A	0.27	0.38	0.56	0.0–0.79	0.11
	MSP3B	0.33	0.24	0.39	0.0–0.63	0.33
IgE	AMA1	0.47	0.62	0.74	0.50–0.96	**0.0001**
	MSP1-19	0.44	0.01	0.11	0.0–0.46	0.57
	MSP2A	0	0.15	0	0.0–0.26	-
	MSP2B	0.62	0.31	0.60	0.0–0.77	0.09
	MSP3A	0.41	0.50	0.45	0.0–0.83	0.21
	MSP3B	0.81	0.10	0.92	0.63–0.96	**0.003**

*r*, Pearson's correlation coefficient; n, number of twin pairs; CI, confidence interval of *h* ^2^ estimate; P values for significant heritability estimates are in bold.

**Table 3:** Pairwise correlations (*r*) and heritability estimates (*h* ^2^) of antibody isotype responses to *P. falciparum* blood stage antigens for child twins (wet season).

Antibody	Antigens	Monozygous *r* (n = 30)	Dizygous *r* (n = 77)	Heritability *h* ^2^	95% CI	P value
IgG1	AMA1	0.71	0.53	0.40	0.0–0.80	0.06
	MSP1-19	0.69	0.35	0.52	0.0–0.76	0.06
	MSP2A	0.47	0.15	0.45	0.0–0.68	0.07
	MSP2B	0.44	0.28	0.30	0.0–0.66	0.44
	MSP3A	0.82	0.33	0.85	0.64–0.91	**0.0001**
	MSP3B	0.78	0.41	0.82	0.48–0.89	**0.0001**
IgG2	AMA1	0.35	0.19	0.64	0.10–0.83	**0.03**
	MSP1-19	0.64	0.11	0.41	0.0–0.62	0.08
	MSP2A	0.45	0.53	0	0.0–0.49	-
	MSP2B	0.41	0.29	0.08	0.0–0.59	0.83
	MSP3A	0.87	0.38	0.84	0.55–0.90	**0.0001**
	MSP3B	0.90	0.49	0.85	0.53–0.94	**0.0001**
IgG3	AMA1	0.59	0.61	0	0.0–0.32	-
	MSP1-19	0.36	0.08	0.32	0.0–0.65	0.22
	MSP2A	0.77	0.62	0.35	0.0–0.69	0.05
	MSP2B	0.64	0.44	0.49	0.0–0.81	0.10
	MSP3A	0.24	0.31	0.34	0.0–0.70	0.40
	MSP3B	0.65	0.47	0.51	0.01–0.82	**0.04**
IgG4	AMA1	0.75	0.40	0.64	0.15–0.83	**0.01**
	MSP1-19	0.81	0	0.96	0.90–0.98	**0.0001**
	MSP2A	0.76	0.43	0.60	0.14–0.83	**0.01**
	MSP2B	0.90	0.45	0.88	0.53–0.94	**0.0001**
	MSP3A	0.70	0.22	0.93	0.84–0.96	**0.0001**
	MSP3B	0.50	0.27	0.52	0.0–0.73	0.07
IgM	AMA1	0.67	0.47	0.44	0.0–0.79	0.10
	MSP1-19	0.06	0	0	0.0–0.24	-
	MSP2A	0	0.32	0	0.0–0.52	-
	MSP2B	0.44	0	0.28	0.0–0.56	0.19
	MSP3A	0.69	0.36	0.71	0.22–0.83	**0.01**
	MSP3B	0.47	0.37	0.58	0.0–0.78	0.10
IgA	AMA1	0.22	0.55	0	0.0–0.41	-
	MSP1-19	0.82	0.04	0.36	0.0–0.56	0.09
	MSP2A	0.56	0.10	0.57	0.11–0.80	**0.02**
	MSP2B	0.64	0	0.38	0.0–0.63	0.06
	MSP3A	0.12	0.16	0.44	0.0–0.74	0.20
	MSP3B	0	0.27	0.49	0.0–0.72	0.30
IgE	AMA1	0.21	0.01	0.14	0.0–0.39	0.50
	MSP1-19	0.28	0.17	0	0.0–0.18	-
	MSP2A	0.64	0.34	0.10	0.0–0.62	0.74
	MSP2B	0.19	0.20	0.48	0.0–0.77	0.18
	MSP3A	0.24	0.59	0	0.0–0.21	-
	MSP3B	0.21	0.48	0	0.0–0.45	-

*r*, Pearson's correlation coefficient; n, number of twin pairs; CI, confidence interval of *h* ^2^ estimate; P values for significant heritability estimates are in bold.

The IgG4 heritability estimates were highest of all isotypes and subclasses, with *h* ^2^ values being above 0.5 for responses to all antigens by the child twins in both dry and wet seasons, indicating that IgG4 responses are most strongly genetically regulated (Figure 1). Analysing the data for each season separately, heritability of IgG4 was significantly higher (P<0.05) than all other isotypes except IgE (P = 0.08) in the dry season, and was significantly higher than all except IgG1 (P = 0.17) and IgG2 (P = 0.14) within the wet season sample. In the adults there were no significant differences in heritability between the different antibody isotypes or subclasses (P values>0.05).

The heritability estimates for IgG4 were lower in the adults than in the children, whether the latter were sampled in the wet or dry season (P = 0.028 for each comparison, Wilcoxon signed ranks tests). Significantly lower heritability in adults was also seen for IgG1 (P = 0.046 compared with children in the wet season) and IgG2 (P = 0.046 compared with children in the dry season). Heritability of IgE levels was significantly lower in the children sampled in the wet season than in the adults or the children sampled in the dry season (P = 0.028 and P = 0.046, respectively).

The significance of variation in HLA class II genes and non-HLA-associated genes to the antibody isotype responses was tested in the adult twin data. The correlations within HLA identical and non-identical DZ twin pairs were compared to test for the contribution of HLA class II genes, whilst the correlations within HLA identical MZ and DZ twin pairs were compared to show the contribution of non-HLA genes. Of the 20 antigen-specific isotype responses with significant overall heritabilities in the adults, 15 (75%) were significantly non-HLA restricted, whereas none showed significant HLA class II restriction (Table 4).

**Table 4:** Contribution of HLA class II and non-HLA genes towards heritability of antibody responses to blood stage antigens in adult twins.

Antibody	Antigens	*r*, HLA identical MZ (n = 58)	*r*, HLA identical DZ (n = 38)	*r*, HLA non-identical MZ (n = 117)	Contribution by non-HLA class II genes *h* ^2^ (95% CI)	Contribution by HLA class II genes *h* ^2^ (95% CI)
IgG1	AMA1	0.70	0.61	0.55	0.18 (0.0–0.65)	0.17 (0.0–0.55)
	MSP1-19	0.61	0.24	0.41	**0.63** (0.24–0.76)	0 (0.0–0.27)
	MSP3A	0.77	0.33	0.35	**0.77** (0.42–0.85)	0 (0.0–0.50)
	MSP3B	0.68	0.37	0.53	**0.68** (0.17–0.80)	0 (0.0–0.24)
IgG2	MSP2A	0.70	0.48	0.39	**0.67** (0.13–0.77)	0 (0.0–0.35)
	MSP3B	0.64	0.05	0.31	**0.55** (0.0–68)	0 (0.0–0.54)
IgG3	AMA1	0.53	0.41	0.34	**0.53** (0.0–0.75)	0.05 (0.0–0.55)
	MSP2A	0.72	0.53	0.35	0.27 (0.0–0.76)	0.33 (0.0–0.68)
	MSP3A	0.62	0.25	0.33	**0.58** (0.0–0.72)	0 (0.0–0.53)
IgG4	AMA1	0.44	0.21	0.11	0.46 (0.0–0.65)	0.28 (0.0–0.55)
	MSP2A	0.58	0.60	0.33	0.11 (0.0–0.66)	0.47 (0.0–0.71)
	MSP2B	0.72	0.02	0.48	**0.73** (0.52–0.83)	0 (0.0–0.16)
	MSP3A	0.58	0.36	0.55	**0.63** (0.14–0.75)	0 (0.0–0.23)
IgA	AMA1	0.55	0.35	0.14	**0.60** (0.03–0.74)	0.30 (0.0–0.51)
	MSP2A	0.65	0.28	0.14	**0.56** (0.01–0.71)	0.28 (0.0–0.49)
	MSP2B	0.54	0.23	0.38	**0.58** (0.16–0.73)	0 (0.0–0.42)
IgE	AMA1	0.43	0.03	0.14	**0.88** (0.78–0.93)	0 (0.0–0.21)
	MSP1-19	0.66	0.29	0	**0.68** (0.43–0.81)	0 (0.0–0.35)
	MSP2B	0.50	0.36	0.28	0.49 (0.0–0.69)	0.54 (0.0–0.71)
	MSP3B	0.47	0.08	0.09	**0.72** (0.44–0.84)	0.01 (0.0–0.35)

*h* ^2^, heritability estimates; *r*, Pearson's correlation coefficient; n, number of twin pairs; CI, confidence interval; MZ, monozygous twins; DZ, dizygous twins; those having a significant contribution by non-HLA genes are shown in bold. Analysis of IgE involved 56 MZ pairs.

## Discussion

This study shows that there are differences in the heritabilities of different antibody isotype and subclass responses to malaria in children, with IgG4 having higher heritability than the other isotypes. There were no significant differences between heritabilities of different antibody isotype levels in the adults, and heritability of IgG4 levels was significantly lower in adults than in children recruited either in the dry season when malaria incidence is low or the wet season when incidence is at an annual peak.

Reasons for higher heritability of IgG4 responses can be considered in the light of different pathways of antibody isotype production. The IgG4 subclass is in relatively low levels in sera, and its production is influenced by IL-4 and IL-13 in human B cells, as also occurs with IgE [38]. However, there were differences between heritabilities for IgG4 and IgE, indicating that they are not solely due to regulation of these cytokines but to other factors. There is evidence that TGFβ and IL-10 produced by regulatory T cells can induce IgG4 production in B cells [26], so genetic variation in regulation of these cytokines may also contribute. The importance of IgG4 responses to malaria remains unclear, as levels of this subclass against malaria antigens are low in all populations including that studied here, although it has been proposed that these antibodies block the binding of effective cytophilic antibodies [15], [20]. It is important to note that the higher heritability of the IgG4 isotype was seen here in both seasonal samples of children, at periods when malaria incidence was highest (wet season) and lowest (dry season), given that serum antibody responses have a limited persistence in young children compared with older children [39] and adults [40]. The heritability estimate was lower in the adult sample (which was not seasonally focused), suggesting that heritability may be more apparent early in life or when acquired immunity is less developed.

The genetic regulation of all antigen-specific isotype responses is shown to be mainly by non-HLA genes, as expected. An earlier analysis of IgG levels to parts of MSP1 and MSP2 had surprisingly not shown the contribution of non-HLA genes to be statistically significant [13]. The heritability of responses to vaccines (BCG, polio, hepatitis B, diphtheria, pertussis and tetanus) in infants has been reported [41], indicating that antibody responses are influenced mainly by non-HLA genes whilst cellular responses to BCG are regulated more by genes within the HLA class II region. It has been shown that although genetic factors control early antibody responses to vaccines, the persistence and avidity maturation of these responses are regulated by environmental factors such as frequent exposure to the microorganisms that influences the subsequent activation of memory B cells [42].

A genome-wide approach is required for the discovery of the genetic determinants of the different antibody isotype responses, as our knowledge of candidate genes remains very limited [3]. For example, several genes in the chromosome region 5q31–q33 region are likely to regulate antibody responses to blood stage malaria infection [43]–[45], including a single nucleotide polymorphism (SNP) at the transcriptional start site of the *IL-4* gene promoter region (*-590C/T*), considered to influence the transcriptional levels of *IL-4* [46]. Studies in Burkina Faso and Ghana indicate that the *IL-4 -590T* allele is associated with higher levels of IgE among severe malaria patients [47], [48], and a study in Mali shows the same allele to be associated with higher levels of *P. falciparum* specific IgE reactivity in the Fulani population, although not in another ethnic group [49]. Surveys in Mali, Gabon and Sudan indicate that variation in plasma IgG2 levels to malaria antigens is non-randomly associated with the amino acid 131 polymorphism of its receptor FcγRIIa that affects binding [50]–[52]. These polymorphisms clearly explain only a small part of the variation in each case, and many others are also likely to have small effects [53].

In summary, this study has shown that genetic factors regulate the different antibody isotype and subclass responses to malaria antigens, mainly due to undefined non-HLA-linked genes. Identification of the genes responsible should be possible by conducting large genome-wide association studies of malaria-exposed individuals with different antibody isotype response profiles [3].
